# Masc‐induced dosage compensation in silkworm cultured cells

**DOI:** 10.1002/2211-5463.12698

**Published:** 2019-07-27

**Authors:** Susumu Katsuma, Keisuke Shoji, Yudai Sugano, Yutaka Suzuki, Takashi Kiuchi

**Affiliations:** ^1^ Department of Agricultural and Environmental Biology, Graduate School of Agricultural and Life Sciences The University of Tokyo Japan; ^2^ Department of Agrobiology and Bioresources, School of Agriculture Utsunomiya University Japan; ^3^ Department of Computational Biology and Medical Sciences, Graduate School of Frontier Sciences The University of Tokyo Kashiwa, Chiba Japan

**Keywords:** *Bombyx mori*, CCCH‐tandem zinc finger protein, dosage compensation, Masc protein, silkworm

## Abstract

The *Masculinizer* (*Masc*) gene encodes a CCCH‐tandem zinc finger protein that controls both masculinization and dosage compensation in the silkworm *Bombyx mori*. We previously measured the masculinizing activity of the lepidopteran Masc proteins using *B. mori* ovary‐derived cell line BmN‐4. Here, we established an RNA‐seq data‐based assay system in which the level of *B. mori* Masc (BmMasc)‐induced dosage compensation can be estimated in BmN‐4 cells. Using this system, we found that a cysteine residue at position 301, which was shown to be essential for the masculinizing activity of BmMasc, is also required for dosage compensation. We further investigated the relationships between Masc‐induced cell growth inhibition, masculinizing activity, and the level of dosage compensation, using *Masc* genes from three lepidopteran insects. In summary, we have established a cell‐based system to monitor levels of Masc‐induced dosage compensation.

**Abbreviations**
DEG: differentially expressed gene
*dsx*: *doublesex*
*IMP*: *IGF‐II mRNA‐binding protein*
*Masc*: *Masculinizer*
piRNA: PIWI‐interacting RNA
ZF: zinc finger

Most animals have an XY sex chromosome system, in which males have X and Y sex chromosomes, whereas females have two X chromosomes. On the other hand, in most lepidopteran insects, females are the heterogametic sex. In this system, which is analogous to the one found in birds and some reptiles, males have two Z sex chromosomes and females have Z and W sex chromosomes. The silkworm *Bombyx mori*, a model species of lepidopteran insects, uses a WZ sex determination system, and the W chromosome is known to possess a dominant role in female determination 1, 2.

Dosage compensation is a common mechanism that equilibrates X‐ or Z‐linked gene expression with the autosomes. In *Drosophila melanogaster*, which uses an XY system, males compensate for reduced dosage of X‐linked genes by hypertranscribing their hemizygous X chromosome through chromosome‐wide hyperacetylation of H4K16 3, 4. *Sex‐lethal*, a master switch for sex determination in *D. melanogaster*, controls dosage compensation by inhibiting translation of *male‐specific lethal 2* (*msl‐2*), and mutations in *msl‐2* lead to male‐specific lethality 5. These findings demonstrate that sex determination cascade is tightly coupled with dosage compensation system and a failure of dosage compensation results in sex‐specific death.

In 2014, we reported that the Z‐linked gene *Masculinizer* (*Masc*) determines maleness in the silkworm *B. mori* (Lepidoptera: Bombycidae) 6. We found that depletion of *Masc* mRNA in early embryos results in abnormal upregulation of Z‐linked genes and male‐specific embryonic death. These results indicate that *Masc* plays essential roles in both masculinization and dosage compensation in *B. mori* embryos 6. In females, *Masc* mRNA is cleaved by the PIWI protein complexed with the W chromosome‐derived female‐specific PIWI‐interacting RNA (piRNA), *Fem* piRNA, which inhibits masculinization and leads to feminization 6.

*Masc* encodes a CCCH‐tandem zinc finger (ZF) protein that is conserved among lepidopteran insects 6, 7, 8, 9, 10. Transfection of *Masc* cDNA in *B. mori* ovary‐derived BmN‐4 cells leads to the expression of male‐type variants of both *B. mori doublesex* (*Bmdsx^M^*) and *B. mori IGF‐II mRNA‐binding protein* (*BmIMP^M^*) 6, 7. Using this system, we attempted to search for functional residues required for the masculinizing activity of Masc and identified two cysteines in the highly conserved region of the lepidopteran Masc proteins, at residues 301 and 304 (Cys‐301 and Cys‐304), both of which are required for the masculinizing activity 7. We also found that two ZFs of the Masc protein are not essential for masculinization in BmN‐4 cells 7. We generated several *Masc* knockout *B. mori* strains using the CRISPR/Cas9 system and confirmed that the two ZFs are not required for either masculinization or dosage compensation in *B. mori* 11. However, due to several technical difficulties, we could not determine which amino acid residues are essential for dosage compensation in *B. mori*.

In the current study, we established a new *in vitro* assay system using RNA‐seq data from BmN‐4 cells transfected with *Masc* derivatives and successfully estimated the level of Masc‐induced dosage compensation in BmN‐4 cells. Using this system, we found that Masc Cys‐301 is required for dosage compensation. In addition, we were also able to detect dosage compensation induced by other lepidopteran Masc proteins. We further investigated the relationships between Masc‐induced cell growth inhibition, masculinizing activity, and the level of dosage compensation.

## Materials and methods

### Insect cells

*Bombyx mori* ovary‐derived BmN‐4 cells were cultured at 27 °C in IPL‐41 medium (Applichem, Darmstadt, Germany) supplemented with 10% FBS 12. *Bombyx mori* embryo‐derived NIAS‐Bm‐M1 (M1) cells 13 were cultured at 27 °C in TC‐100 medium (Applichem) supplemented with 10% FBS.

### Plasmids and transfection

The pIZ/V5‐His vectors containing *Fem* piRNA‐resistant *Masc* (*Masc‐R*), *C301S Masc‐R* (*MR‐CS*), *Ostrinia furnacalis Masc* (*OfMasc*), or *Trilocha varians Masc* (*TvMasc*) have been previously described 6, 7, 8, 9. BmN‐4 and M1 cells were transfected with empty or *Masc* plasmids using X‐tremeGENE HP (Roche, Basel, Switzerland). For stable transfection, zeocin (Invitrogen, Waltham, MA, USA, a final concentration of 500 μg·mL^−1^) was added to the medium three days after transfection 14.

### Reverse transcription polymerase chain reaction (RT‐PCR)

Total RNA was prepared from transfected cells using TRIzol reagent (Invitrogen) and used for reverse transcription using avian myeloblastosis virus reverse transcriptase with an oligo‐dT primer (TaKaRa, Kusatsu, Japan). PCR was performed using KOD FX‐neo DNA polymerase (TOYOBO, Osaka, Japan). Sex‐specific splicing of *Bmdsx* was examined with RT‐PCR as reported previously 7. Quantitative RT‐PCR (RT‐qPCR) of *BmIMP^M^* and *rp49* was performed using a KAPA™ SYBR FAST qPCR kit (Kapa Biosystems, Wilmington, MA, USA), as previously described 7.

### RNA‐seq

Libraries for RNA‐seq were generated from total RNA of transfected cells using the SureSelect Strand‐Specific RNA library Prep Kit (Agilent, Santa Clara, CA, USA). The cDNAs were analyzed using the Illumina HiSeq 2500 platform (Illumina, San Diego, CA, USA) with 100‐bp paired‐end reads according to the manufacturer's protocol. RNA‐seq reads were mapped to 13 789 *B. mori* gene models (putative protein‐coding genes whose chromosomal locations are identified), and the transcript abundance in each gene model was quantified as previously described 6, 15.

### Caspase assay

BmN‐4 cells transfected with pIZ/V5‐His vectors containing *Masc* cDNAs were collected 5 days after transfection. Caspase activity was measured using Caspase‐Glo3/7 assay kit (Promega, Fitchburg, WI, USA).

### Sequence deposition

The deep sequencing data obtained in this study are available under the accession number DRA008403 in the DNA Data Bank of Japan (DDBJ).

## Results

### Establishment of a cell‐based assay system for monitoring the Masc protein‐induced dosage compensation

**Figure 1:**
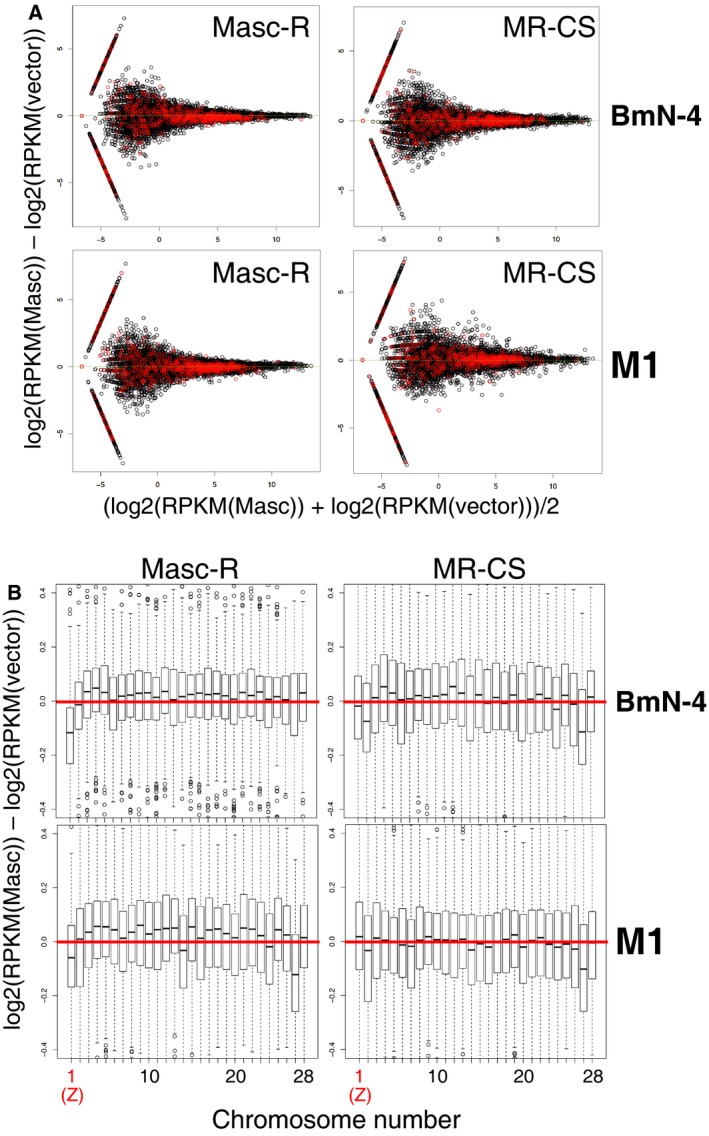
A cell‐based assay system for monitoring the levels of Masc‐dependent dosage compensation using silkworm cultured cells. (A) MA plots of RNA‐seq data of BmN‐4 or M1 cells transfected with empty vector or *Masc* cDNAs. The Z‐linked genes are indicated by red dots. The axes show the following: *A* (*x*‐axis) = [log_2_(transcripts per million in *Masc* cDNA‐transfected cells) + log_2_(transcripts per million in empty vector‐transfected cells)]/2; and *M* (*y*‐axis) = log_2_(transcripts per million in *Masc* cDNA‐transfected cells) − log_2_(transcripts per million in empty vector‐transfected cells). (B) Chromosomal distribution of differentially expressed transcripts in BmN‐4 (upper) or M1 (lower) cells transfected with *Masc‐R* or *MR‐CS* cDNA. The genes whose transcript abundance was higher than the median were used to make the boxplots. The data are shown by box‐and‐whisker diagrams. The boxes represent the median and 25th–75th percentile ranges of the expression ratios.

To estimate the level of dosage compensation governed by the Masc protein, we attempted to establish a cell‐based assay system using *B. mori* cultured cells. We performed RNA‐seq on two *B. mori* cell lines, BmN‐4 and M1, both of which were transfected with *B. mori Masc* (*BmMasc*) or its derivative cDNAs. BmN‐4 is an ovary‐derived female cell line 12, and M1 is an embryo‐derived male cell line 13. We first used two *BmMasc* cDNAs, *Fem* piRNA‐resistant *BmMasc* (*Masc‐R*) and *Cys‐301 to Ser BmMasc‐R* (*MR‐CS*) 5, 7. Because Cys‐301 is completely conserved among lepidopteran Masc proteins and plays an essential role for the masculinizing activity of BmMasc in BmN‐4 cells 7, 10, we speculated that this cysteine residue is also required for dosage compensation. We measured the expression levels of *B. mori* genes in *BmMasc* cDNA‐transfected cells by mapping RNA‐seq reads onto the *B. mori* gene models. MA plots and boxplot of the mapping results clearly show that transfection of *Masc‐R* cDNA decreased the expression of Z‐linked genes (red dots in Fig. 1A) in both BmN‐4 and M1 cells and that the suppression was more apparent in BmN‐4 cells than in M1 cells (Fig. 1A,B). The expression levels of autosomal genes (black dots in Fig. 1A) were comparable in *Masc‐R* cDNA‐transfected cells to those in empty vector‐transfected cells. On the other hand, transfection of *MR‐CS* cDNA did not greatly affect the expression of autosomal and Z‐linked genes in either BmN‐4 or M1 cells (Fig. 1A,B). Combining these observations with our previous results 7, 10, we concluded that Cys‐301 is crucial for both masculinization and dosage compensation of the BmMasc protein. In addition, we observed that the genes on the chromosome 27 were relatively downregulated in M1 cells by transfection of either *Masc‐R* or *MR‐CS* cDNA (Fig. 1B). However, in BmN‐4 cells, transfection of *MR‐CS* cDNA, but not *Masc‐R* cDNA, reduced their expression levels (Fig. 1B), indicating that this effect does not depend on the Masc activity. Taken together, we decided to use BmN‐4 cells to monitor the levels of Masc‐induced dosage compensation.

### Comparison of the levels of dosage compensation induced by three lepidopteran Masc proteins

**Figure 2:**
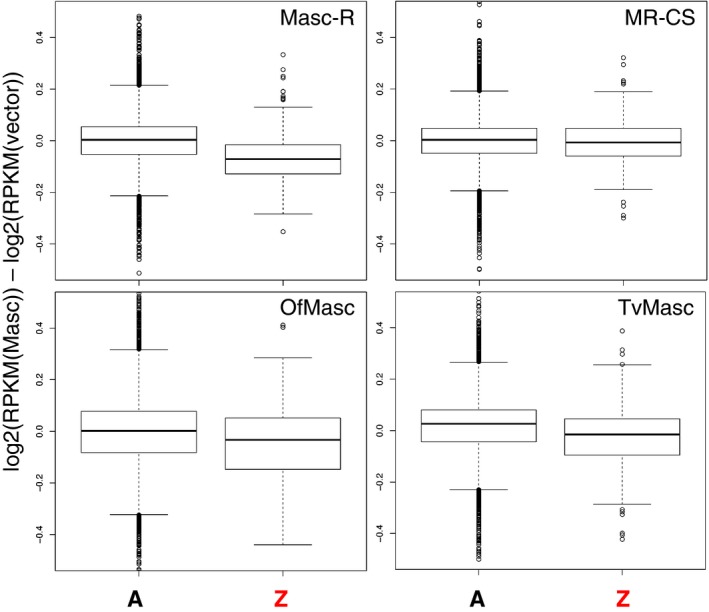
The levels of dosage compensation in BmN‐4 cells transfected with lepidopteran *Masc* cDNAs. Differentially expressed transcripts of the autosomal (A) and Z‐linked (Z) genes in BmN‐4 cells transfected with lepidopteran *Masc* cDNAs. RNA‐seq data were obtained in three independent experiments, and the average values of three experiments were used for analysis. The genes whose average transcript abundance was higher than the median were used to make the boxplots. The data are shown by box‐and‐whisker diagrams. The boxes represent the median and 25th–75th percentile ranges of the expression ratios.

We have previously shown that two Masc proteins, OfMasc from *O. furnacalis* (Lepidoptera: Crambidae) and TvMasc from *T. varians* (Lepidoptera: Bombycidae), exhibit the masculinizing activity in BmN‐4 cells 8, 9. We next assessed the levels of dosage compensation induced by these non‐*BmMasc* proteins using a cell‐based assay system. We performed RNA‐seq of total RNA prepared from BmN‐4 cells transfected with *Masc‐R*, *MR‐CS*, *OfMasc*, and *TvMasc* cDNAs and compared the expression levels of Z‐linked and autosomal genes with those of empty vector‐transfected cells. We reproducibly observed dosage compensation in *Masc‐R*‐transfected cells and little effect on gene expression in *MR‐CS*‐transfected cells (Fig. 2). We also detected repression of Z‐linked genes, but not autosomal genes, in *OfMasc*‐ and *TvMasc*‐transfected cells, indicating that these two non‐*BmMasc* proteins also induce dosage compensation in BmN‐4 cells. The repression levels of Z‐linked genes in *OfMasc*‐ and *TvMasc*‐transfected cells were comparable to those observed in *BmMasc*‐transfected cells (Fig. 2). These results strongly suggest that OfMasc and TvMasc proteins are involved in dosage compensation of Z‐linked genes.

### Cell growth inhibition induced by the Masc proteins

**Figure 3:**
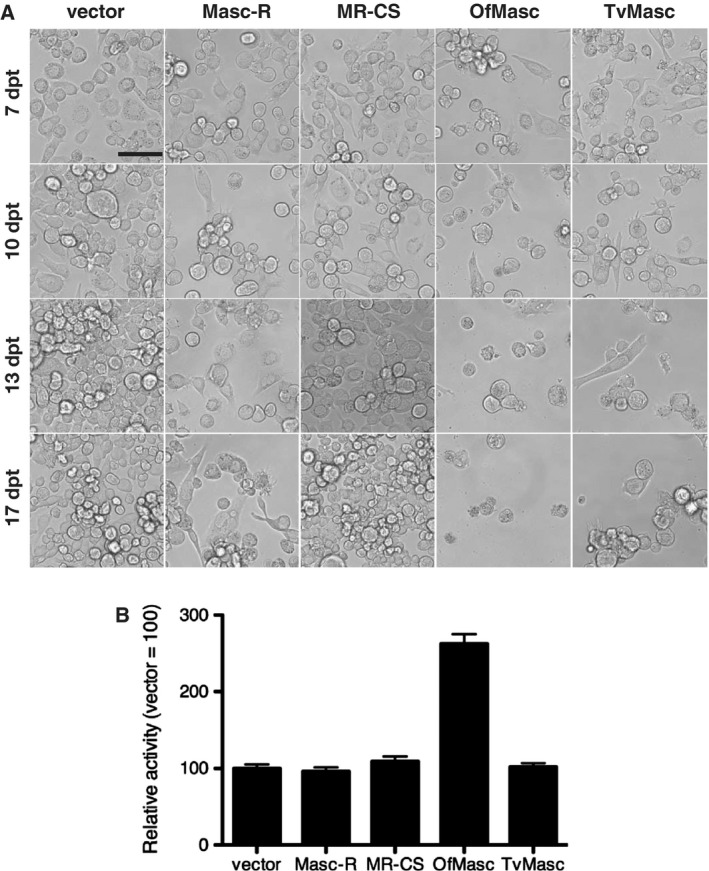
Viability of BmN‐4 cells transfected with *Masc* cDNAs. (A) Light microscopic observations of BmN‐4 cells stably transfected with *Masc* cDNAs. BmN‐4 cells were transfected with empty pIZ/V5‐His vector, pIZ/V5‐His containing *Masc‐R*, *MR‐CS*, *OfMasc*, or *TvMasc*. Three days after transfection, zeocin was added to the medium. Photographs were taken at seven, 10, 13, and 17 days post‐transfection (dpt). Bar: 100 μm. (B) Caspase activity. BmN‐4 cells were transfected with empty vector or *Masc* cDNAs. At five dpt, caspase activity was measured using Caspase‐Glo3/7 assay kit. Data represent the mean ± standard deviation.

In previous studies, we observed that transfection of *Masc‐R* cDNA resulted in growth inhibition in BmN‐4 cells, whereas transfection of *MR‐CS* cDNA did not 6, 10. However, the mechanism of Masc‐induced cell growth inhibition remains unknown. We next assessed the effect of transfection of *OfMasc* or *TvMasc* cDNA on cell growth of BmN‐4 cells. As observed in *Masc‐R* cDNA‐transfected cells, expression of both *OfMasc* and *TvMasc* inhibited cell growth (Fig. 3A). The degree of growth inhibition decreased as follows, from highest to lowest: (a) *OfMasc*‐transfected cells; (b) *Masc‐R*‐ or *TvMasc*‐transfected cells; and (c) *MR‐CS‐* or empty vector‐transfected cells. To examine whether this growth inhibition is associated with the apoptotic pathway, we measured caspase activity in *Masc* cDNA‐transfected cells. As shown in Fig. 3B, the caspase activity was higher in *OfMasc* cDNA‐transfected cells than in cells transfected with other *Masc* cDNAs or empty vector, indicating that the significant inhibition of cell growth observed in *OfMasc*‐transfected cells is caused by apoptosis. Caspase activity in *Masc‐R* and *TvMasc* cDNA‐transfected cells was comparable to that in empty vector‐transfected cells. Although caspase activity in transfected cells may be linked to the expression levels of the Masc proteins, this suggests that cell growth inhibition induced by these two Masc proteins is not associated with apoptosis.

### Comparison of the masculinizing activities of the three Masc proteins

**Figure 4:**
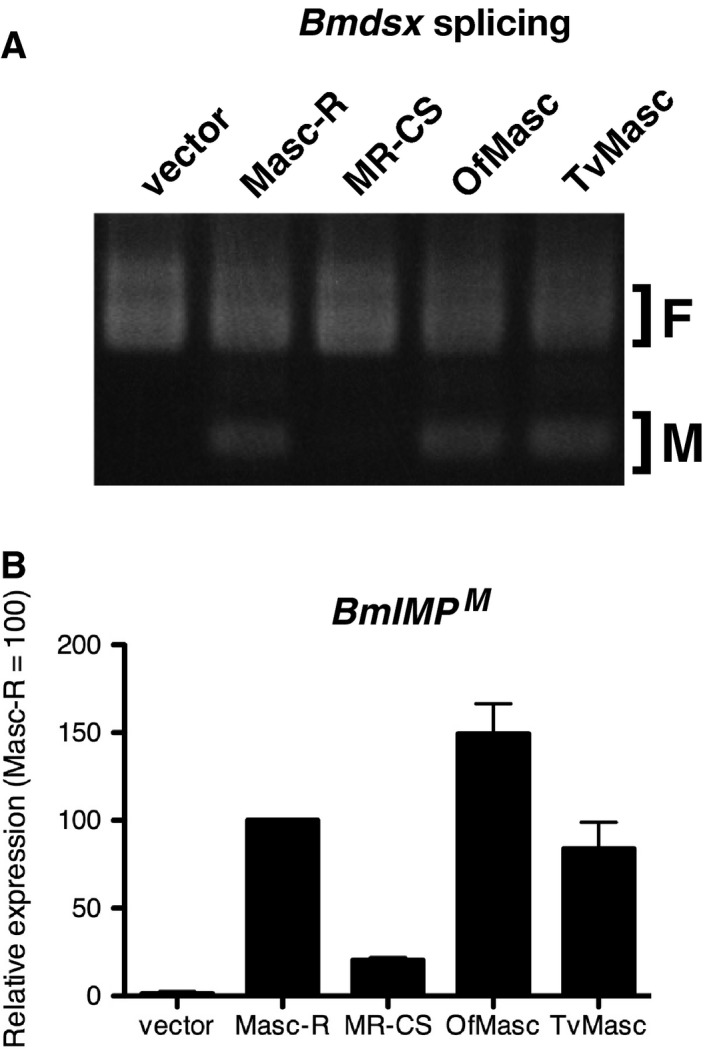
Comparison of the masculinizing activity of the lepidopteran Masc proteins. (A) Splicing pattern of *Bmdsx* in BmN‐4 cells transfected with *Masc‐R*, *MR‐CS*, *OfMasc*, or *TvMasc* cDNAs. *Bmdsx* splicing was examined by RT‐PCR. F and M indicate female‐ and male‐type splicing of *Bmdsx*, respectively. (B) Expression of *BmIMP^M^* in BmN‐4 cells transfected with *Masc‐R*, *MR‐CS*, *OfMasc*, or *TvMasc* cDNAs. The mRNA levels of *BmIMP^M^* were examined by RT‐qPCR. The *BmIMP^M^* mRNA level was normalized to that of *rp49*. The data are shown as means + SD of three independent experiments.

To compare the masculinizing activity of the lepidopteran Masc proteins, we investigated the splicing patterns of *Bmdsx* and the expression levels of *BmIMP^M^* in *Masc* cDNA‐transfected cells. As previously reported 5, 6, 7, 8, 9, transfection of *Masc* cDNAs from three lepidopteran species induced the expression of *Bmdsx^M^* and *BmIMP^M^* (Fig. 4A,B). The band intensities of *Bmdsx^M^* were similar among the three Masc proteins, whereas the level of *BmIMP^M^* expression was ~ 1.5 times higher in *OfMasc* cDNA‐transfected cells than in cells transfected with other *Masc* cDNAs (Fig. 4B).

## Discussion

In this study, we successfully established a cell‐based system to monitor the levels of Masc‐induced dosage compensation. Using this new system, we identified Cys‐301 of BmMasc as an essential residue for both dosage compensation and masculinization and were able to estimate the levels of dosage compensation induced by non‐*BmMasc* proteins. These results indicate that this system is capable of identifying residues or domains of the Masc proteins involved in dosage compensation, and of assessing the levels of dosage compensation induced by other lepidopteran Masc proteins. In addition, our findings strongly suggest that both roles of the Masc protein are conserved not only in the closely related species (*T. varians*) but also in the evolutionarily distant species (*O. furnacalis*).

We reported that transfection of *Masc‐R* cDNA results in severe inhibition of cell growth in BmN‐4 cells 6 and that transfection of *MR‐CS* cDNA did not induce cell growth inhibition 10, indicating that BmMasc activity is required for this inhibition. In this study, we compared the levels of masculinization and dosage compensation in BmN‐4 cells transfected with three lepidopteran *Masc* cDNAs and found that Masc‐induced growth inhibition appears to be associated with its masculinizing activity (Fig. 4B). In addition, we showed that transfection of *OfMasc*, but not *BmMasc* or *TvMasc*, induces caspase activation, although growth inhibition was observed in BmN‐4 cells transfected with each *Masc* cDNA. To know this reason, we attempted to identify differentially expressed genes (DEGs) using RNA‐seq data from *Masc* cDNA‐transfected cells. However, we did not identify DEGs common to all treatments or apoptosis‐related DEGs in *OfMasc* cDNA‐transfected cells (data not shown). Further experiments, such as analyzing RNA‐seq data from samples taken at different time points after transfection, are needed to clarify the mechanism of Masc‐induced cell growth inhibition in BmN‐4 cells.

Recently, Zheng *et al*. reported that BmMasc induces the A and B isoforms of the RNA‐binding protein gene *BxRBP3*, whose overexpression in female cultured cells (BmF cells) efficiently inhibits splicing of the exons 3 and 4 in *Bmdsx* and enhances *Bmdsx^M^* expression 16. To confirm whether this signaling pathway is also utilized in BmN‐4 cells, we examined the expression of *BxRBP3* isoforms in BmN‐4 cells using RNA‐seq data. We found that the A and B isoforms were neither induced by transfection with *Masc* cDNAs nor detected in the transcriptome of BmN‐4 cells (data not shown). RNA‐seq analysis showed that the D isoform of *BxRBP3* is predominant in BmN‐4 cells. RT‐PCR using published primers confirmed the results obtained from our RNA‐seq data (data not shown), suggesting that Masc‐induced masculinization signaling differs between BmN‐4 and BmF. We are currently searching for the factors that are activated under the Masc signaling in silkworm cells.

## Conflict of interest

The authors declare no conflict of interest.

## Author contributions

SK designed the study. SK and YS performed molecular biological experiments. KS performed most of the bioinformatics analyses. YS obtained and analyzed the RNA‐seq data. SK, KS, YS, and TK analyzed the experimental data. All of the authors discussed the data and helped in manuscript preparation. SK wrote the manuscript with intellectual input from all authors.
